# Risk of Malignant Transformation of Giant Cell Tumors of Bone Is 8 Times Lower with Megavoltage vs. Orthovoltage Radiation Therapy

**DOI:** 10.1155/2022/7216296

**Published:** 2022-10-21

**Authors:** Farah N. Musharbash, Alexander Edelstein, Jad M. El Abiad, Adam S. Levin, Sara R. Alcorn, Carol D. Morris

**Affiliations:** ^1^Department of Orthopaedic Surgery, The Johns Hopkins University School of Medicine, Baltimore, Maryland, USA; ^2^Department of Orthopaedic Surgery, Guthrie Robert Packer Hospital, Sayre, Pennsylvania, USA; ^3^Department of Orthopaedic Surgery, American University of Beirut, Beirut, Lebanon; ^4^Department of Oncology, The Johns Hopkins University School of Medicine, Baltimore, Maryland, USA; ^5^Department of Radiation Oncology, The Johns Hopkins University School of Medicine, Baltimore, Maryland, USA

## Abstract

**Background:**

The first-line treatment for most giant cell tumors (GCTs) of bone is surgical; radiotherapy (RT) is reserved for inoperable or refractory cases. While RT techniques have undergone a dramatic change over the past few decades, with the higher energy megavoltage RT replacing orthovoltage RT, concerns for high rates of malignant transformation following RT have limited its use. Evidence suggests a lower incidence of secondary malignancy after treatment with megavoltage compared with orthovoltage RT, but this has not been studied in GCTs. Our main purpose was to compare the incidence of malignant transformation of GCTB between patients treated with orthovoltage vs. megavoltage RT.

**Methods:**

A literature review was performed to identify studies reporting GCTBs treated with RT from 01/1900 through 12/2019. Studies that did not report RT modality or separate orthovoltage and megavoltage results were excluded. Included in the analysis were 6 patients from our institution. Primary outcome was the incidence of malignant transformation; secondary outcomes were time to transformation and incidence of local recurrence. Fisher's exact tests and independent sample *t*-tests were used, and significance was set at *p* < 0.05.

**Results:**

Twenty-two studies were included, which reported on 168 GCTBs treated with orthovoltage and 393 treated with megavoltage RT. Transformation incidence was 14% (*n* = 24) for orthovoltage and 1.8% (*n* = 7) for megavoltage RT, an 8-fold difference (odds ratio (OR) 9.1, 95% confidence interval (CI) 3.9–22, *p* < 0.001). Mean time to transformation was 8.7 years for orthovoltage and 11.2 years for megavoltage RT (*p*=0.28). Incidence of local recurrence was 38% (63/167) for orthovoltage and 17% (66/393) for megavoltage RT (OR 3.3, 95% CI 2.0–4.6, *p* < 0.001).

**Conclusions:**

The risk of developing a malignancy after RT of GCTB is 8 times lower with megavoltage than with orthovoltage. Malignant transformation with megavoltage, while not zero, is lower than that in historical series. Use of modern RT techniques in inoperable or refractory GCTB may be appropriate.

## 1. Introduction

Giant cell tumor (GCT) of bone is a benign yet aggressive lesion that comprises approximately 5% of primary adult bone tumors [1]. Approximately 1% to 2% of patients with GCT of bone develop hematogenous metastasis to the lungs [2]. GCTs of bone are primarily treated surgically when they are accessible in a low-morbidity anatomic location. Radiotherapy has typically been reserved for surgically unresectable or refractory GCTs and is associated with risk of malignant or sarcomatous transformation, limiting its routine use [3, 4].

During the past several decades, however, external-beam radiotherapy technology has improved dramatically. Orthovoltage radiotherapy, which uses X-ray beams with an energy of 150–500 kV, has been replaced by megavoltage radiotherapy, which uses linear accelerators, Van de Graaf generators, or cobalt teletherapy units to produce X-rays and gamma rays with an average energy greater than 1 million electron volts [5]. Previous research in pediatric tumors has suggested that the incidence of secondary malignant neoplasms may be higher after orthovoltage radiotherapy compared with megavoltage radiotherapy; however, we are aware of no studies addressing this question specifically in GCTs of bone [6, 7].

The primary purpose of this study was to compare the incidence of malignant or secondary transformation of GCTs of bone after radiotherapy with orthovoltage vs. megavoltage. We hypothesized that modern megavoltage radiotherapy would be associated with a lower incidence of malignant transformation.

## 2. Materials and Methods

### 2.1. Study Selection

A literature review was conducted in December 2019 using PubMed, Embase, Cochrane Library, Web of Science, and Scopus. The terms “giant cell tumor of bone,” “giant cell,” “osteoclastoma,” “radiotherapy,” and “radiation” and their combinations were used to identify studies in which radiotherapy was used to treat GCTs of bone. The titles and abstracts of the results of the search were reviewed by 2 authors to identify relevant articles. Full texts of relevant articles were subsequently reviewed to determine whether they met our inclusion and exclusion criteria. References of the articles chosen for inclusion were also reviewed for any relevant articles. Any disagreements were discussed and resolved by the senior author.

**Figure 1:**
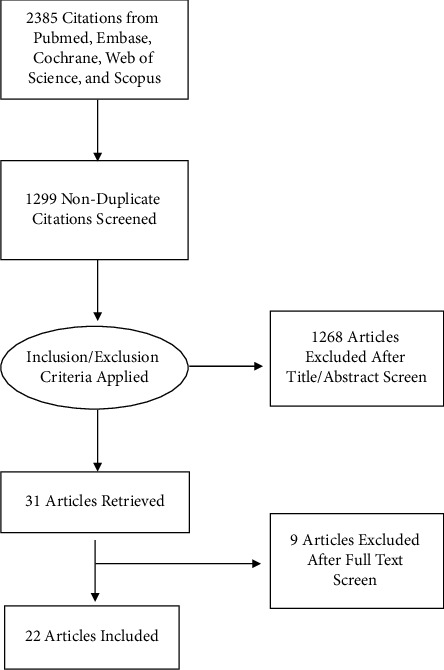
PRISMA flowchart showing the literature search and study selection process.

Studies were included if they reported outcomes of radiotherapy for patients with benign GCTs of bone (*n* = 2385) (Figure 1). Studies published in languages other than English and those not involving human patients were excluded. After duplicates were removed (*n* = 1086), 1299 study titles/abstracts were reviewed, and 31 full texts were read in more detail, with 9 more excluded for missing or indifferentiable data (unable to differentiate or infer whether orthovoltage vs. megavoltage therapy was used) (Figure 1). Five studies reported orthovoltage radiotherapy only, 14 reported megavoltage only, and 3 reported both. Twenty-two studies were included in our analysis, in addition to 6 patients from our institution [3, 4, 8–27].

#### 2.1.1. Study Population

A total of 168 patients with GCTs underwent treatment with orthovoltage radiotherapy and 393 patients with GCTs underwent treatment with megavoltage radiotherapy. Patient age at diagnosis ranged from 7 to 67 years in the orthovoltage group and from 11 to 85 years in the megavoltage group. Patient, radiation, and tumor characteristics are summarized in Table 1.

## 3. Data Extraction

This study was approved by our institutional review board (IRB00174908). The following data were extracted from each study: type of radiotherapy used, number of GCTs treated, incidence of malignant or sarcomatous transformation, time to transformation, and incidence of local recurrence. Patients who had a recurrence with malignant or sarcomatous transformation were included in the local recurrence incidence. Other data that were collected but could not be analyzed because of inconsistencies in reporting were patient age, sex, follow-up duration, radiation dosage, histology of secondary malignancy, time to local recurrence, and whether the radiation was adjuvant or primary. Data on patients from our institution were obtained from electronic medical records. Data were extracted by 2 authors and reviewed by a third author.

### 3.1. Statistical Analysis

The primary outcome measure was the difference in incidence of malignant transformation of benign GCTs after orthovoltage vs. megavoltage radiotherapy. Odds ratios were calculated for both malignant transformation and local recurrence. Fisher's exact tests were used to compare the odds of transformation and recurrence between orthovoltage and megavoltage groups, and independent samples *t*-tests were used to compare the mean time to transformation between groups. Significance was set at *p* < 0.05. Statistical analysis was performed using Stata Statistical Software: Release 14 (StataCorp LLC, College Station, TX).

**Figure 2:**
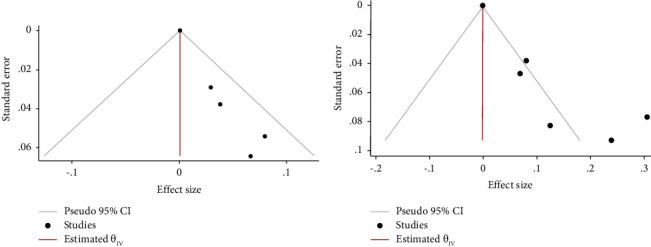
Funnel plots for studies in the megavoltage group (a) and the orthovoltage group (b).

Cochran's *q* test was performed to evaluate the heterogeneity of studies within the orthovoltage and megavoltage groups. Cochran's *q* test for heterogeneity demonstrated a *p* value of <0.01 for the orthovoltage studies, indicating high heterogeneity, and a *p* value of 0.978 for the megavoltage studies, demonstrating low heterogeneity. A funnel plot was created for each group to investigate publication bias, and these are shown in Figure 2. All studies in the megavoltage group, compared with 5 of the 8 studies in the orthovoltage group, were within the 95% CI bounds of the funnel plot.

## 4. Results

The incidence of malignant or sarcomatous transformation was 14% (*n* = 24) in the orthovoltage group and 1.8% (*n* = 7) in the megavoltage group, an 8-fold difference. There was a significant between-group difference in the incidence of transformation (odds ratio (OR) 9.1, 95% confidence interval (CI) 3.9–22, *p* < 0.001).

Mean time to transformation was 8.7 years in the orthovoltage group and 11.2 years in the megavoltage group (*p*=0.28).

The incidence of local recurrence was 38% (63/167) in the orthovoltage group and 17% (66/393) in the megavoltage group. The difference in the risk of local recurrence was statistically significant between the 2 groups (OR 3.3, 95% CI 2.0 to 4.6, *p* < 0.001).  Table 2 summarizes the details of malignant/sarcomatous transformation and local recurrence for each study.

In studies in which management was reported, most patients with malignant or sarcomatous transformation were managed with amputation alone (67% (14/21)). This was followed by non-ablative surgery alone in 10% (2/21) and non-ablative surgery with radiation therapy in 10% (2/21) of cases. Radiation therapy alone, radiation therapy with chemotherapy, and amputation with radiation therapy were each reported once each as the mode of management of malignant or sarcomatous transformation. For all patients with transformation, the mortality was 75% (21/28).

## 5. Discussion

Despite major advances in radiotherapy technology, there is hesitancy for treating GCTs of bone with radiotherapy. This hesitancy may be caused by the historically high association of radiotherapy with malignant or sarcomatous transformation [28, 29]. Although benign, GCTs can be locally aggressive and can cause substantial morbidity. Most of the studies reporting a higher association between radiotherapy and malignant or sarcomatous transformation were published during the era of orthovoltage radiotherapy.

Our results indicate that the risk of developing of malignant or sarcomatous transformation is 8 times lower in patients treated with megavoltage radiotherapy compared with orthovoltage radiotherapy (1.8% vs. 14.3%), a difference that is statistically significant. Although we believe ours to be the first study showing this difference for GCTs of bone, prior studies have suggested a lower incidence of post-irradiation sarcomas and malignant transformation in patients treated with megavoltage radiotherapy compared with orthovoltage radiotherapy, including pediatric patients [6, 7]. It is interesting to note that malignant transformation can also occur without radiotherapy. In a study by Campanacci et al. [2], 2 of 280 patients (0.71%) developed a secondary malignancy after undergoing only surgical treatment. Similarly, Dahlin et al. [24] reported on 195 patients with GCT of bone, of whom 2 (1.0%) developed a secondary malignant transformation after surgical treatment only. In a recent pooled analysis of 4 large case series studying malignant GCTs, approximately one-quarter (14/56) of secondary malignancies occurred after surgery without treatment with radiotherapy [30]. This finding suggests that the risk of developing a secondary malignancy from radiotherapy with megavoltage beams may be lower than originally thought, especially when compared with the risk with surgery as sole treatment. Our results also indicate that the incidence of local recurrence is approximately twice as high for orthovoltage radiotherapy as for megavoltage radiotherapy (38% vs. 17%). It has been suggested that orthovoltage radiotherapy is more harmful to bone tissue because of the photoelectric effect at the lower energy range of orthovoltage radiotherapy, wherein bone tissues absorb a 2 to 3-fold higher radiation dose than the surrounding soft tissues. In contrast, with the higher energy range of megavoltage radiation, the Compton scatter effect predominates, leading to a uniform dose to bone and soft tissue [30, 31]. This may help explain the higher incidence of malignant transformation seen with orthovoltage radiotherapy.

Our study has several limitations. First, we were unable to include all studies reporting the results of radiotherapy for GCTs of bone because some did not report the type of radiotherapy received. Second, there was significant heterogeneity among the orthovoltage studies. Despite the funnel plot suggesting publication bias for orthovoltage studies, the asymmetry is likely secondary to the heterogeneity of the studies as opposed to true publication bias because these studies were case series rather than comparative studies. Nevertheless, the underlying heterogeneity is a limitation of the current investigation. Third, follow-up durations for each patient in the series were not available in many studies, which prevented us from performing a Kaplan–Meier analysis and from comparing follow-up periods for each group because of variations in reporting. Recognizing that an increased latency period might result in increased numbers of malignancy reported, it is possible that a difference in the follow-up period between the two groups may contribute to some of the difference seen in the rates of malignant transformation.

Another factor we were unable to evaluate for because of missing data was the cumulative radiation dose for each patient. In addition, while management of local recurrence included repeat excision, radiation therapy, and chemotherapy in some cases, many studies did not report the management or outcomes after local recurrence, making meaningful analysis of such outcomes difficult. Another potential limitation is the way malignant or sarcomatous transformation was documented for each patient. In 27 cases, this was confirmed histologically; in two cases in the study by Bradshaw [25], malignancy was diagnosed through local behavior and metastasis, and in two other cases, the means of confirmation of malignancy was not documented. Finally, a confounding factor that could not be addressed is the advances in surgical treatment between the older orthovoltage era and the more recent megavoltage era. Better surgical protocols and preoperative imaging may be confounding factors leading to the lower incidence of local recurrence observed with megavoltage therapy. However, the large magnitude of the difference in incidence of malignant or sarcomatous transformation between orthovoltage radiotherapy and megavoltage radiotherapy makes the difference less likely to be the result of differences in concomitant surgical techniques or advances in imaging compared with the difference in the incidence of local recurrence.

We recognize that, in the current era, denosumab has a prominent role in achieving local control of GCT in difficult anatomic regions. Denosumab is not without its own controversy as it pertains to the relationship between benign and malignant tumors. Although the purpose of this study was not to compare the indications, efficacy, or limitations of radiotherapy vs. denosumab, we believe radiotherapy deserves reconsideration in the contemporary management of refractory or surgically unresectable GCT of bone; however, further studies comparing it to denosumab for such indications may be warranted.

## 6. Conclusion

The historically high incidence of malignant transformation of benign GCTs after radiation exposure seems largely attributable to orthovoltage radiotherapy. Our results indicate an 8 times higher risk of malignant or sarcomatous transformation with orthovoltage radiotherapy compared with megavoltage radiotherapy, and approximately twice the risk of local recurrence. These findings suggest that radiotherapy of GCTs of bone may be safer than previously thought. Although some may believe that any increase in risk of malignant transformation is unacceptable for benign conditions, radiotherapy for GCT of bone warrants further consideration, especially for refractory or surgically unresectable disease.

## Figures and Tables

**Table 1:** Patient, radiation, and tumor characteristics of the included studies.

First author (year)	No. of tumors	Mean (range) age at diagnosis, yr	Range of radiation dose, Gy	Radiation only (*n*)	Radiation timing (*n*)		GCTB (*n*)		
					Preoperative	Postoperative	Primary	Recurrent	Metastatic
*Megavoltage*									
Ruka [16] (2010)	77	28 (16–69)^*∗*^	26–89	77	0	0	56	21	0
Bhatia [17] (2011)	58	31 (12–84)^*∗*^	20–65	13	0	42	45	13	0
Shi [14] (2013)	34	29 (16–85)^*∗*^	35–55	21	0	13	22	12	0
Sharma [11] (1990)	30	27 (18–40)	45–55	30	0	0	30	0	0
Feigenberg [13] (2003)	26	35 (16–75)	35–55	15	0	11	16	10	0
Caudell [4] (2003)	25	32 (11–69)^*∗*^	25–65	14	0	11	13	12	0
Malone [15] (1995)	21	32 (13–71)	11–50	7	0	14	13	8	0
Chakravati [8] (1999)	20	39 (15–72)	40–70	7	0	13	15	4	1
Nair [3] (1999)	20	33 (15–65)	40–60	20	0	0	14	6	0
Bennett [10] (1993)	16	35 (16–75)	35–54	16	0	0	10	6	0
Bell [9] (1983)	15	28 (13–49)	33–55	15	0	0	11	4	0
Schwartz [19] (1989)	13	45 (15–63)	42–68	8	0	5	7	3	3
Seider [20] (1986)	9	34 (12–63)	36–66	1	0	8	9	0	0
Daugaard [12] (1987)	8	38 (22–62)	30–75	0	1	7	6	2	0
JHH	6	48 (31–60)	50–60	0	0	6	2	4	0
Khan [18] (1999)	6	47 (16–71)	30–54	1	0	5	6	0	0
Roeder [21] (2010)	5	37 (20–60)	57–64	3	0	2	3	2	0
Walter [22] (1960)	4	35 (16–49)	30–44	3	0	1	4	0	0

*Orthovoltage*									
Bradshaw [25] (1964)	50	NA (7–66)	20–60	23	8	19	50	0	0
Dahlin [24] (1970)	36	NA	NA	7	0	29	7	29	0
Windeyer [26] (1949)	29	NA (8–62)	2000–6000^†^	19	0	10	29	0	0
McGrath [27] (1972)	21	NA	3000–9500^†^	12	0	9	16	5	0
Mnaymneh [23] (1964)	16	31 (12–44)	2400–5400^†^	6	0	10	9	7	0
Walter [22] (1960)	12	39 (17–67)	20–44	11	1	0	12	0	0
Daugaard [12] (1987)	2	36 (35–37)	23–24	0	0	2	2	0	0
Seider [20] (1986)	2	35 (34–36)	2100–4500^†^	0	0	2	2	0	0

GCTB, giant cell tumor of bone; JHH, Johns Hopkins Hospital patients; NA, not available. ^*∗*^Expressed as median (range). ^†^Unit of measure is Roentgen.

**Table 2:** Details of malignant/sarcomatous transformation and local recurrence for each study.

First author (year)	No. of tumors	Mean (range) follow-up, yr	Malignant/sarcomatous transformation (*n*)	Mean (range) time to transformation, yr	Local recurrence (*n*)	Mean (range) time tolocal recurrence, yr
*Megavoltage*						
Chakravati [8] (1999)	20	9.3 (NA)^*∗*^	0		3	0.69 (0.4–1)
Bell [9] (1983)	15	12 (2–25)	1	21 (NA)	1	2 (NA)
Bennett [10] (1993)	16	9 (NA)	0		4	15 (0.7–2.1)
Sharma [11] (1990)	30	9.2 (NA)	0		2	7.7 (7–8.3)
Daugaard [12] (1987)	8	6.5 (NA)	0		1	0.7 (NA)
Feigenberg [13] (2003)	26	12 (2.4–26)	1	22 (NA)	6	1.2 (1.1–1.7)
Shi [14] (2013)	34	17 (1.4–34)^*∗*^	1	4.3 (NA)	6	1.9 (0.1–4.4)^*∗*^
Malone [15] (1995)	21	15 (2–35)	0		2	2.5 (1–4)
Ruka [16] (2010)	77	4.8 (0.5–33)^*∗*^	2	4 (3.4–4.6)	12	2.3 (0.3–6.3)^*∗*^
Nair [3] (1999)	20	5.3 (NA)	0		2	1.3 (1.1–1.5)
Bhatia [17] (2011)	58	8 (4–28)^*∗*^	0		8	∼2.5 (NA)
Caudell [4] (2003)	25	10 (NA)	2	11.5 (11–12)	10	0.9 (NA)^*∗*^
Khan [18] (1999)	6	13 (NA)	0		1	3 (NA)
Schwartz [19] (1989)	13	6.5 (1.5–13)	0		2	0.7 (0.4–1)
Seider [20] (1986)	9	11 (NA)	0		3	2.7 (0.5–6)
Roeder [21] (2010)	5	4.9 (NA)	0		1	0.25 (NA)
Walter [22] (1960)	4	2.6 (2–4)	0		1	0.5 (NA)
JHH	6	14 (10–21)	0		1	0.3 (NA)

*Orthovoltage*						
Mnaymneh [23] (1964)	16	31 (12–44)	2	3.4 (1.7–5)	14	2.4 (0.2–5)
Walter [22] (1960)	12	8.2 (2–20)	0		2	1 (0.5–1.5)
Dahlin [24] (1970)	36	NA	11	9 (3.7–38)	17	<3 (NA)
Bradshaw [25] (1964)	50	>5 (NA)	4	NA (1.5–8.5)	16	NA
Windeyer [26] (1949)	29	NA	2	NA	4	NA
McGrath [27] (1972)	21	NA	5	10 (1–17)	8	NA
Seider [20] (1986)	2	24 (18–30)	0	0	0	
Daugaard [12] (1987)	2	12 (8–16)	0	0	2	1 (0.2–1.8)

JHH, Johns Hopkins Hospital patients; NA, not available. ^*∗*^Expressed as median (range).

## Data Availability

Most data used for this study can be found on PubMed, Embase, Cochrane Library, Web of Science, and Scopus. Exception is for the 6 patients from our institutional database which may be provided upon request.
